# The effects of clonal integration and earthworms on the growth, active constituent accumulation in *Glechoma longituba*, and the soil microbial community in its root zone

**DOI:** 10.3389/fpls.2025.1596905

**Published:** 2025-09-15

**Authors:** Bing-Nan Zhao, Xiao-Gai Wang, Rui Zhang, Xue-Ge He, Zi-Yang Xie, Xiao-Qin Yao, Chao Si

**Affiliations:** ^1^ College of Life Sciences, Hebei University, Baoding, China; ^2^ School of Life Science and Engineering, Handan University, Handan, China

**Keywords:** active constituent, clonal integration, clonal plant, *Glechoma longituba*, soil microorganism community

## Abstract

**Introduction:**

Clonal integration represents a key ecological advantage of clonal plants, enabling resource transfer among interconnected ramets. Earthworm activity significantly influences soil processes and plant growth. However, it remains unclear whether the effects of earthworms on basal ramets can be transmitted to their apical counterparts through clonal integration.

**Methods:**

We conducted an experiment using *Glechoma longituba*, a commonly used clonal herb. Basal ramets were either exposed or not exposed to earthworms (*Pheretima guillelmi*) in the soil, while apical ramets remained devoid of earthworm presence. The stolon connecting the ramets was either severed or left intact.

**Results:**

Clonal integration promoted the growth of apical ramets at the expense of basal ramets, resulting in no net gain or loss at the whole fragment level. Although the direct effect of earthworms on plant growth was minimal, they interact with clonal integration to affect the number of nodes and total stolon length. Clonal integration enhanced the accumulation of total flavonoids in the whole fragment, whereas earthworms had a significant negative effect on the accumulation of chlorogenic acid. Clonal integration significantly affected bacterial composition at both basal and apical portions, while concurrently reducing bacterial diversity. Earthworms accumulated specific fungal communities and increased fungal diversity at apical portions. Earthworms also reduced the difference within bacterial communities in the basal portions of both intact and severed treatments to some extent.

**Conclusion:**

Our findings indicate that clonal integration and earthworms influence the accumulation of active constituents in G. longituba at the whole fragment level, without significantly promoting plant growth. Moreover, earthworms alter the bacterial and fungal communities in the root zone of apical ramets via clonal integration. These results provide a theoretical foundation for the cultivation of this species.

## 1 Introduction

Clonal integration, a prominent characteristic of clonal plants, facilitates the sharing of resources (i.e., water, mineral nutrients and photosynthates) and signals among ramets through interconnected structures such as stolons and rhizomes (Hutchings and de Kroon, 1994; Kroon, 1993; Liu et al., 2007; Song et al., 2013). For younger ramets, clonal integration typically enables them to acquire resources from older interconnected ramets, thereby enhancing their survival and growth (Alpert, 1996; Zhang et al., 2003). In cases where ramets encounter poor resource availability or unfavorable environmental conditions, interconnected ramets located in resource-rich or favorable environments can supply essential resources through clonal integration, thus improving the fitness of the stressed ramets (Li et al., 2024; Si et al., 2020; Zhang et al., 2023). Furthermore, this resource transfer establishes a cost-benefit relationship between recipient and donor ramets (Roiloa and Retuerto, 2011; Zhang et al., 2023; Zhao et al., 2024). Previous studies have shown inconsistent results regarding whether clonal integration leads to a net benefit or cost at the whole fragment level, which largely depends on whether it imposes a cost on donor ramets (Roiloa and Retuerto, 2011; Si et al., 2020; Cao et al., 2022). In some studies, donor ramets remained unaffected or even gain benefit from compensatory responses to stress experienced by recipient ramets, despite providing them with resources (Xu et al., 2010; Song et al., 2013; Wang et al., 2021; Cao et al., 2022). Conversely, other studies have demonstrated that donor ramets may suffer growth reductions due to the diversion of resources to recipient ramets (Roiloa and Retuerto, 2011; Si et al., 2020; Zhao et al., 2024). These discrepancies may be attributed to variations in species characteristics and the environmental factors involved in the respective studies. Therefore, further experimental evidence is necessary to clarify the effects of clonal integration.

Earthworms, prevalent in diverse terrestrial ecosystems, are commonly introduced into agricultural soils to enhance soil quality (Boyer and Wratten, 2010; Capowiez et al., 2006; Jouquet et al., 2014). They significantly improve soil structure, fertility, and overall health by creating channels that increase aeration and water infiltration, both critical for plant growth (Mcinerney et al., 2001; Xiao et al., 2019). Their burrowing activity mixes organic matter deeper into the soil profile, enhancing nutrient availability for plants (Rombke et al., 2005; Shuster et al., 2001). Earthworms also transform dead leaves and plant residues into nutrient-rich castings via their digestive processes, thereby supplying essential nutrients like nitrogen, phosphorus, and potassium (Araujo et al., 2004; Lavelle et al., 1992; Rombke et al., 2005). Furthermore, earthworms play a crucial role in maintaining soil microbial diversity, fostering a sustainable farming environment (Gong et al., 2019, Gong et al., 2023; Wang et al., 2024b). However, it remains unclear whether earthworms can interact with clonal integration to influence plant growth and physiological processes.

Beyond earthworms, soil microorganisms also maintain close interactions with plants, exerting reciprocal influences on one another (Pang et al., 2009; Wang et al., 2024c, Wang et al., 2024b). In clonal plants, when clonal integration occurs, alterations in growth and physiological activities of interconnected ramets due to resource translocation through clonal integration may concurrently influence root growth, root litter production, and root exudates release. These changes may further affect the growth dynamics, community structure, and functional attributes of soil microorganisms within the root zone. Although previous studies have shown that in heterogeneous light environments, clonal integration altered the rhizosphere microbial community structure and composition of shaded ramets in *Glechoma longituba* and *Phyllostachys nigra* (Lei et al., 2014; Zhang and Chen, 2017), there is still limited available evidence to fully comprehend the effects of clonal integration on soil microorganisms, particularly those in the root zone of clonal plants, remains limited.

We conducted a greenhouse experiment to investigate the effects of clonal integration and earthworms on the growth, accumulation of bioactive constituents, and soil microbial community in the root zone of *G. longituba*, a commonly clonal plant in China. We planted pairs of interconnected ramets of *G. longituba* in two adjacent pots, with or without the addition of earthworms into the soil where the basal ramets (relatively older) were rooted; no earthworms were added to the soil where the apical ramets (relatively younger) were rooted. Furthermore, the stolons connecting the basal and apical ramets were left intact or severed. Specifically, we aimed to test the following hypotheses: (i) clonal integration affects the growth, bioactive constituent contents, and root zone microbial communities in both basal and apical ramets; (ii) earthworms added to the basal ramets can affect the growth, bioactive constituent contents, and root zone microbial communities of the apical ramets through clonal integration.

## 2 Materials and methods

### 2.1 Plant and earthworm species

*Glechoma longituba* is a typical clonal herb belonging to the Lamiaceae family (Jin et al., 2019a; Wei et al., 2020; Zuo et al., 2015). It rich in various active constituents such as chlorogenic acid and flavonoids compounds, making it valuable for medicinal use (Grabowska et al., 2022; Jin et al., 2019b; Wang et al., 2024a; Zhang et al., 2023). This species exhibits monopodial stolons with nodes that have the potential to give rise to new ramets (Chu et al., 2006; Quan et al., 2021; Xing et al., 2024). It has a wide distribution across China, excluding Qinghai, Gansu, Xinjiang, and Tibet (Chu et al., 2006; Zhang et al., 2007). The plants of *G. longituba* used in this experiment were purchased from a commercial supplier in Shanghai. Prior to the commencement of the experiment, the plants were cultivated for several weeks in a greenhouse (36°34′N, 114°29′E) at Handan University in Handan, Hebei Province, China.

The earthworm species *Pheretima guillelmi* Michaelsen (Megascolecinae) predominantly inhabits deep soils and primarily feeds on the litter layer, occasionally ingesting soil particles (Lowe and Butt, 2002; Meng et al., 2022; Rombke et al., 2005). This species typically measures between 15 to 25 cm in length with a body width ranging from 5 to 8 mm (Lowe and Butt, 2002; Rombke et al., 2005). It exhibits a wide distribution across southern China. Recently, *P. guillelmi* has been utilized for manure digestion and soil quality improvement due to its pivotal role in enhancing plant growth through regulation of soil microorganisms (Chang et al., 2023; Si et al., 2021; Dempsey et al., 2013; Hoang et al., 2017; Zeb et al., 2020; Zheng et al., 2015). The earthworms used in this study were purchased from a commercial supplier in Jurong, Jiangsu Province, China.

### 2.2 Experimental design

On 11 May 2022, more than a hundred ramets (each consisting of one node and a pair of leaves) were cut from the stock plants. These ramets were cultivated in seed tray filled with a substrate composed of vermiculite (particle size was 1–2 mm) and potting soil (pH: 5.5-6.5, organic matter: > 45%; Jiqing Biotechnology Co. LTD, China) at a volume ratio of 1:1. On 19 May 2022, twenty well-grown fragments of equal size (comprising 3–5 new ramets and an apex) were selected for this experiment.

The three relatively older ramets, referred to as the basal portion, were centrally rooted in pots measuring 18 cm in diameter and 15.5 cm in height. The apex and its adjacent ramet, referred to as the apical portion, were allowed to root in separate pots of identical size placed nearby. Each pot was filled with a mixture of potting soil (7.9 g total N kg^-1^ and 224.7 g total C kg^-1^; Dewoduo Fertilizer Co., China) and locally collected topsoil (0.83 g total N kg^-1^ and 20.37 g total C kg^-1^) at a volume ratio of 1:1. On 24 May 2022, after confirming successful colonization of all plants, ten pairs of *G. longituba* were randomly selected for severed treatment by cutting the stolons that connected the basal and apical portions in the middle, thereby dividing the fragment into independent basal and apical portions. The stolons of the rest plants were left intact so that their basal and apical portions remained interconnected.

**Figure 1:**
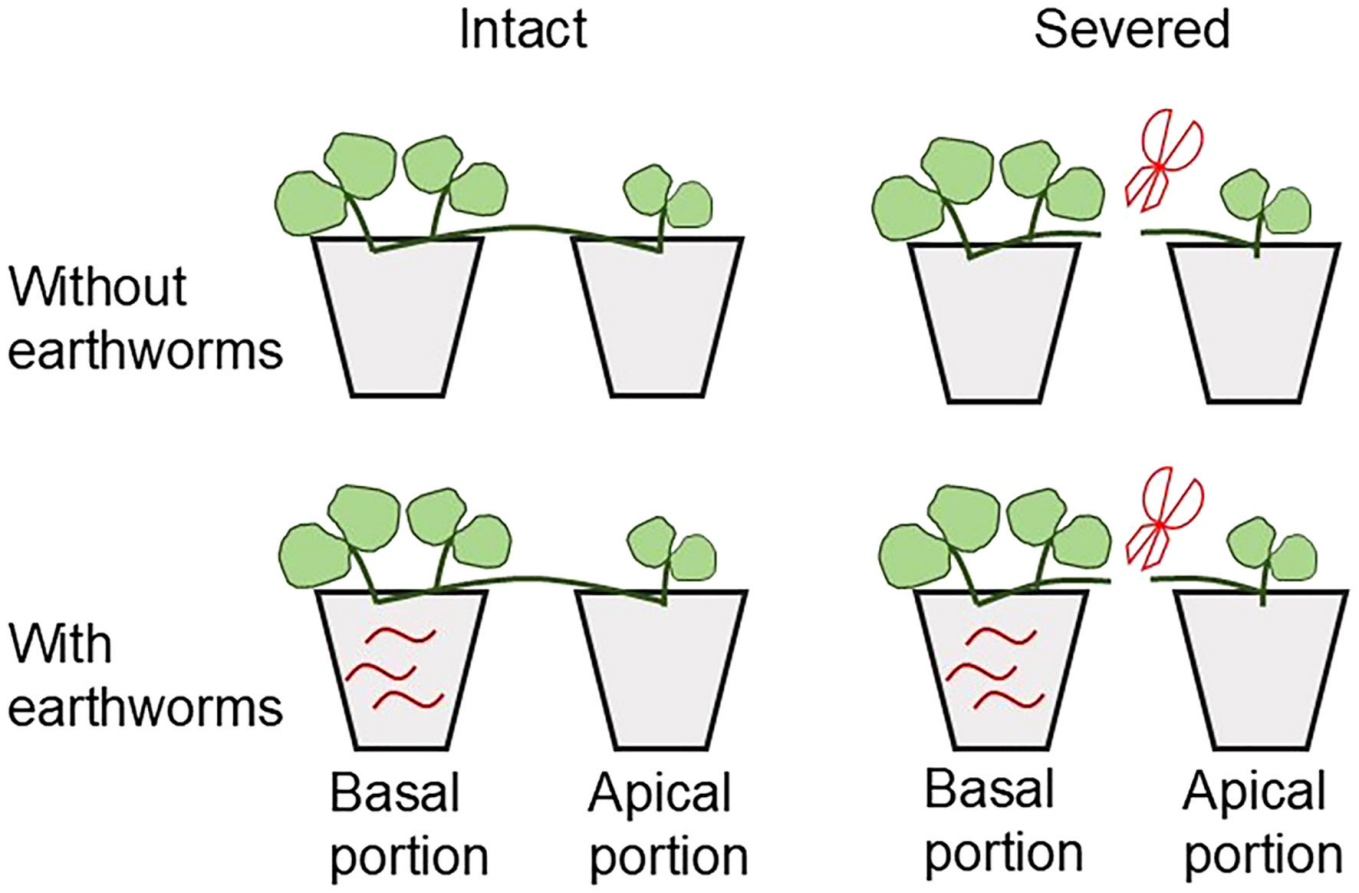
Schematic representation of the experimental design.

For the earthworm treatment, three earthworms (representing common arable soil densities; Cao et al., 2006; Tao et al., 2009) were introduced exclusively into the soil of basal portions. No earthworms were introduced into apical portions in any treatment group. To compensate for potential earthworm loss caused by their mobility during the experiment, three additional earthworms were introduced on 3 June, 2022, 18 June, 2022, and 3 July, 2022 respectively. All pots were randomly placed on a bench within the same greenhouse for plant cultivation. Newly generated ramets from each portion were allowed to root in their respective original pots. The experimental design is illustrated in **Figure 1**.

The experiment was conducted until 18 July 2022. Throughout the experimental period, the air temperature and humidity in the greenhouse were measured every 2 hours using a temperature logger, yielding mean values of 29.7°C and 53.5%, respectively. During the experiment, the soil in the pots was kept moist by daily watering.

### 2.3 Measurements and data analysis

At harvest, we carefully removed the soil matrix adhered to the roots of plants in each pot, and counted both the number of nodes and total stolon length. Subsequently, the plants were divided into leaves, stolons, and roots, dried at 70°C until a constant weight was achieved, and weighed. We also calculated the specific stolon length (total stolon mass/total stolon length) as well as the root-shoot ratio [root mass/(leaf mass + stolon mass)]. The determination of total flavonoids and chlorogenic acid content was conducted using spectrophotometry with slight modifications to the method described by previous studies (Li et al., 2019; Liu et al., 2021).

After harvesting the plants, we collected soil from each pot and removed visible debris, dirt, and plant roots. Subsequently, the soil samples were thoroughly mixed and stored at -40°C for analysis of the microbial community in the root zone. Total genomic DNA of soil microorganisms was extracted using the FastDNA SPIN kit (MP Biomedicals, USA) according to the manufacturer’s instructions. The V4 region of the bacterial 16S rRNA gene was amplified using the primers 515F (5’-GTGYCAGCMGCCGCGGTAA-3’) and 806R (5’-GACTACNVGGGTWTCTAAT-3’) (Walters et al., 2016). For amplification of the ITS1 region of the fungal internal transcriptional spacer (ITS), the primers ITS1f (5’-CTTGGTCATTTAGAGGAAGTAA-3’) and ITS2 (5’-GCTGGT TCTTCATCGATGC-3’) were employed (Walters et al., 2016). Paired-end sequencing of the DNA fragments was conducted on the Illumina Novaseq platform at Shanghai Personal Biotechnology Co., Ltd., Shanghai, China.

Analysis of variance (ANOVA) tests were performed to examine the effects of clonal integration and earthworms on a series of measurements about plant growth, bioactive constituent content, and soil microbial community in this experiment. Prior to analysis, all data were checked for homoscedasticity and transformed as necessary to improve homoscedasticity. Specific data transformation methods are provided in the tables. All statistical analyses were performed using SPSS 22.0 (IBM, Inc, ArInonk, NY, USA). Figures show untransformed data. Chlorogenic acid and total flavonoid content measurements could not be obtained in two replicates (one replicate involved severed stolons with earthworms, while the other replicate involved intact stolons without earthworms) due to insufficient sample amount; therefore, these samples were excluded from further analyses.

In bioinformatics analyses, raw sequence data were filtered and underwent preliminary processing using QIIME2 (Bolyen et al., 2019). The Dada2 method was utilized to trim adapter sequences and primers from raw reads, remove low-quality sequences and chimeras, denoise, and assemble the sequences. Deduplicated sequences were grouped into Amplicon Sequence Variants (ASVs). Singleton ASVs were excluded during the subsequent analytical steps. Bacterial taxonomies were assigned by aligning against the Silva database (Glöckner et al., 2017), while fungal taxonomies were assigned using the UNITE database (Nilsson et al., 2015). Ultimately, the bacterial and fungal ASV tables were resampled to achieve a uniform count of 50,239 sequences per sample for bacteria and 51,430 sequences per sample for fungi, respectively.

In QIIME2, the Chao1 index was calculated to assess the alpha diversity of bacterial and fungal communities. Venn diagram showed the distribution of bacterial and fungal ASV under different treatments. Principal coordinate analysis (PCoA) was employed to visualize the beta diversity among treatments. Indicator species with significantly different relative abundances among treatments were identified using LEfSe analysis. The importance score of microbial species on microbial community changes was assessed using random-forest analysis. To assess the associations between microbial communities and plant factors, as well as the pairwise relationships among the plant factors themselves, we performed Mantel tests and Pearson correlation analyses, visualizing correlation strength and significance. The above analyses were performed by the genescloud tools, a free online platform for data analysis (https://www.genescloud.cn).

## 3 Results

### 3.1 Effects of clonal integration and earthworms on growth and morphology of the basal portion, apical portion and the whole clonal fragment

**Figure 2:**
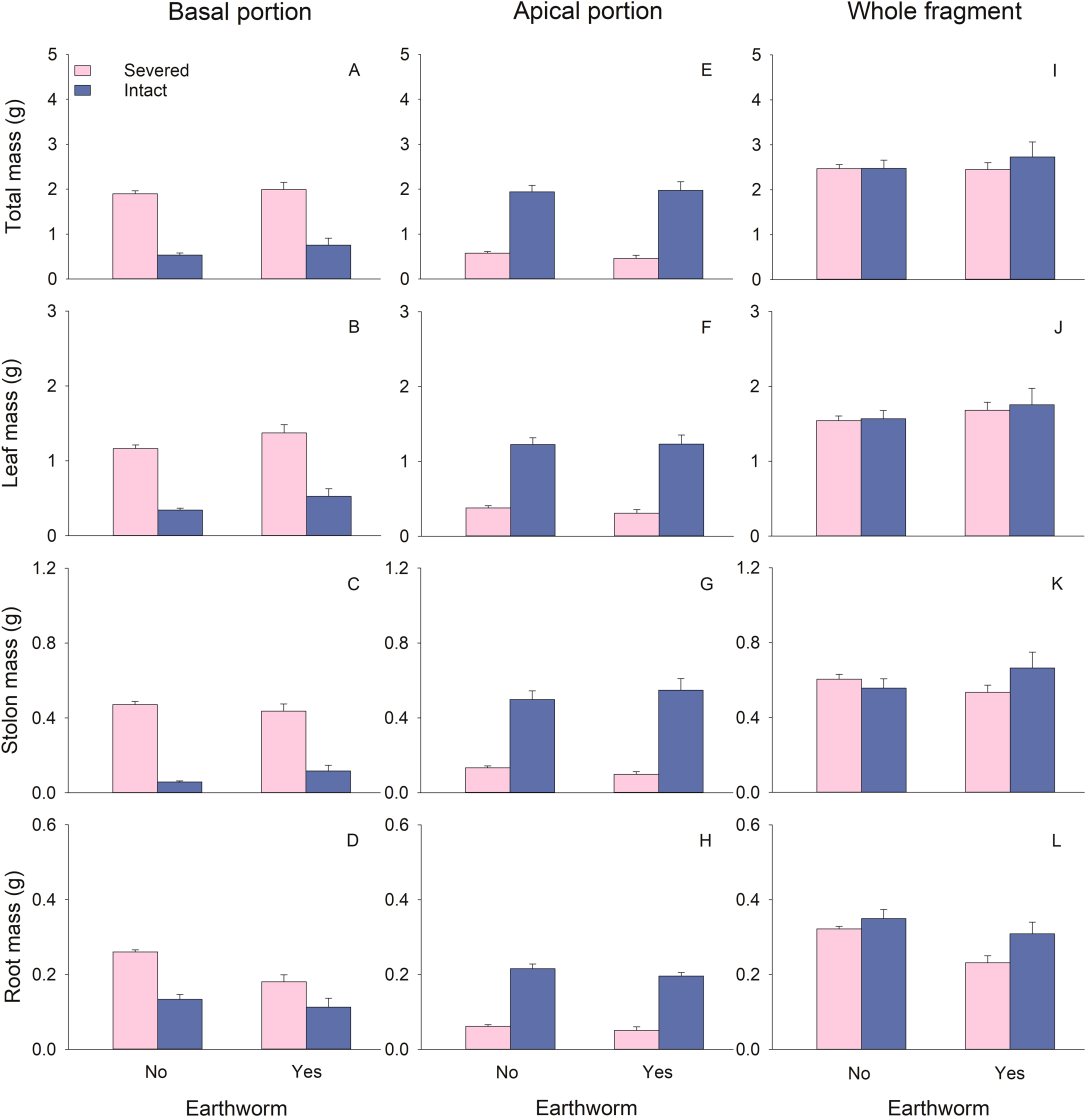
Effects of clonal integration and earthworms on total mass **(A, E, I)**, leaf biomass **(B, F, J)**, stolon biomass **(C, G, K)**, and root biomass **(D, H, L)** of the basal portion, apical portion, and the whole fragment of *Glechoma longituba*. Bars and vertical lines represent mean and SE (n = 5).

**Figure 3:**
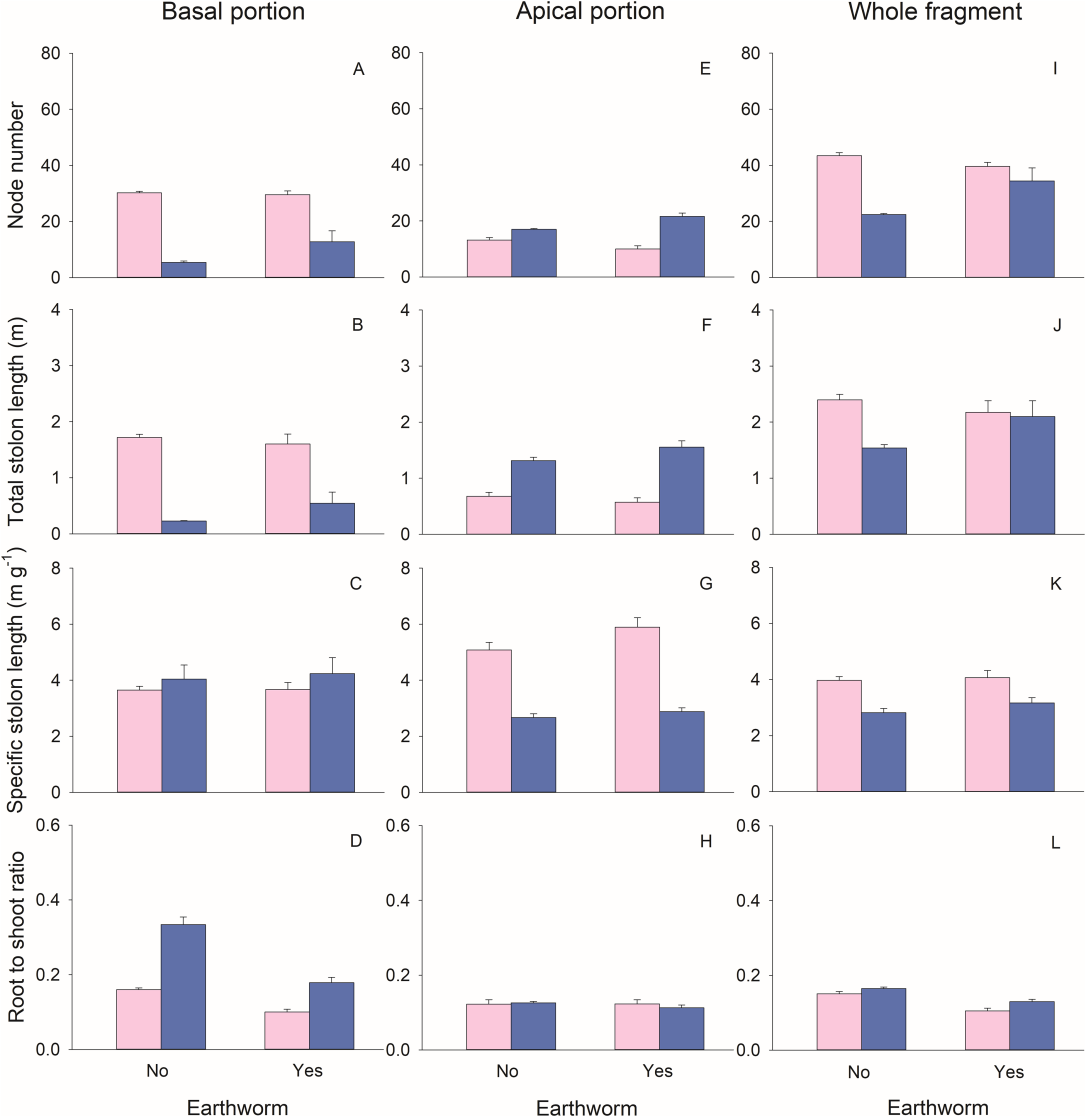
Effects of clonal integration and earthworms on node number **(A, E, I)**, total stolon length **(B, F, J)**, specific stolon length **(C, G, K)**, and root-shoot ratio **(D, H, L)** of the basal portion, apical portion, and the whole fragment of *Glechoma longituba*. Bars and vertical lines represent mean and SE (n = 5).

The biomass (total, leaf, stolon, and root), node number, and total stolon length of basal portions of *G. longituba* were significantly reduced when stolons were left intact compared to when they were severed (**Table 1**; **Figures 2A-D**, **3A, B**). However, the root-shoot ratio significantly increased when stolons were left intact (**Table 1**; **Figure 3D**). Stolon treatment had no effect on specific stolon length (**Table 1**; **Figure 3C**). The presence of earthworms resulted in higher leaf mass but lower root mass and root-shoot ratio compared to treatments without earthworms (**Table 1**; **Figures 2B, D**, **3D**). The interaction between stolon treatment and earthworm treatment had no significant effect on any measurements of plant growth or morphology in the basal portions (**Table 1**).

**Table 1:** Analysis of variance of the effects of clonal integration, earthworms, and their interaction on growth and morphology of basal portion, apical portion and the whole fragment of *Glechoma longituba*.

Variable	Integration (I)		Earthworm (E)		I × E	
	F_1,16_	*P*	F_1,16_	*P*	F_1,16_	*P*
Basal portion						
Total mass	**119.1**	**< 0.001**	1.7	0.205	0.3	0.602
Leaf mass	**105.0**	**< 0.001**	**5.8**	**0.029**	< 0.1	0.875
Stolon mass	**197.2**	**< 0.001**	0.2	0.660	3.2	0.093
Root mass	**34.0**	**< 0.001**	**9.0**	**0.008**	3.1	0.095
Node number	**101.2**	**< 0.001**	2.7	0.120	3.7	0.071
Total stolon length	**87.2**	**< 0.001**	0.6	0.464	2.5	0.131
Specific stolon length ^a^	0.8	0.375	< 0.1	0.856	< 0.1	0.840
Root to shoot ratio ^a^	**109.1**	**< 0.001**	**78.9**	**< 0.001**	1.5	0.233
Apical portion						
Total mass ^b^	**177.3**	**< 0.001**	0.6	0.457	0.9	0.360
Leaf mass ^b^	**160.5**	**< 0.001**	0.6	0.439	0.6	0.449
Stolon mass ^b^	**170.3**	**< 0.001**	0.1	0.711	2.2	0.160
Root mass	**250.4**	**< 0.001**	2.6	0.125	0.2	0.641
Node number ^b^	**61.3**	**< 0.001**	< 0.1	0.897	**15.5**	**0.001**
Total stolon length ^b^	**89.8**	**< 0.001**	< 0.1	0.759	3.8	0.068
Specific stolon length ^a^	**172.6**	**< 0.001**	**4.7**	**0.045**	0.5	0.499
Root to shoot ratio ^a^	< 0.1	0.818	0.5	0.487	0.7	0.413
Whole clonal fragment						
Total mass a	0.3	0.614	0.2	0.688	0.3	0.569
Leaf mass ^a^	< 0.1	0.849	1.2	0.299	< 0.1	0.959
Stolon mass ^a^	0.3	0.579	< 0.1	0.868	2.7	0.118
Root mass	**5.6**	**0.031**	**8.7**	**0.009**	1.3	0.274
Node number ^a^	**38.6**	**< 0.001**	**5.1**	**0.038**	**13.4**	**0.002**
Total stolon length ^a^	**7.9**	**0.012**	0.9	0.367	**4.8**	**0.044**
Specific stolon length ^b^	**28.8**	**< 0.001**	1.4	0.253	0.6	0.442
Root to shoot ratio ^b^	**10.3**	**0.006**	**44.6**	**< 0.001**	0.8	0.380

Degrees of freedom (d. f.), F and *P* values are given. Values are in bold when *P*<0.05. ^a^Natural log transformation. b Square root transformation.

For the apical portions, biomass, node number, and total stolon length were significantly higher when stolons were left intact compared to when they were severed (**Table 1**; **Figures 2E-H**, **3E, F**). However, specific stolon length was significantly reduced when stolons were left intact (**Table 1**; **Figure 3G**). Stolon treatment had no significant effect on the root-shoot ratio (**Table 1**; **Figure 3H**). The presence of earthworms significantly increased specific stolon length but did not affect other measurements of apical portions (**Table 1**; **Figure 3G**). The interaction between stolon treatment and earthworm treatment only had a significant effect on node number (**Table 1**). Specifically, when stolons were left intact, earthworms significantly increased node number, whereas this effect was not observed when stolons were severed (**Figure 3E**).

At the whole fragment level, intact stolons treatment exhibited higher root mass and root-shoot ratio compared to severed stolons treatment (**Table 1**; **Figure 2L**, **3L**). However, node number, total stolon length, and specific stolon length significantly decreased when stolons were left intact (**Table 1**; **Figures 3I-K**). Stolon treatment had no significant effect on total mass, leaf mass, or stolon mass of the whole clonal fragment (**Table 1**; **Figures 2I–K**). In the presence of earthworms, root mass and root-shoot ratio were reduced, while node number increased compared to treatments without earthworms (**Table 1**; **Figure 2L**, **3I, L**). The interaction between stolon treatment and earthworm treatment significantly affected node number and total stolon length (**Table 1**). Specifically, when stolons were left intact, the presence of earthworms increased node number and total stolon length, an effect that was not observed when stolons were severed (**Figures 3I, J**).

### 3.2 Effects of clonal integration and earthworms on content of the total flavonoids and chlorogenic acid in the basal portion, apical portion and the whole clonal fragment

**Figure 4:**
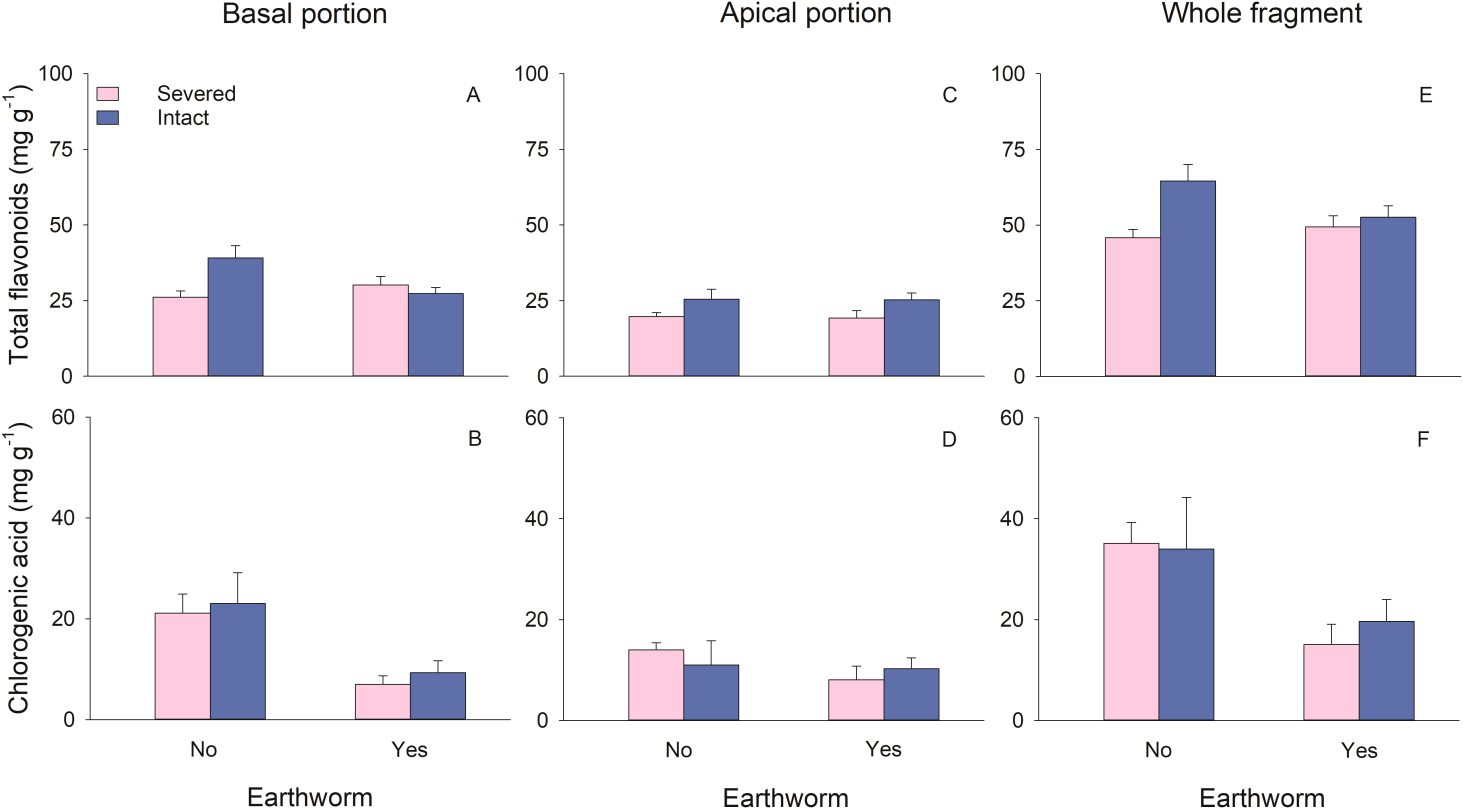
Effects of clonal integration and earthworms on total flavonoids **(A, C, E)** and chlorogenic acid **(B, D, F)** of the basal portion, apical portion, and the whole fragment of *Glechoma longituba*. Bars and vertical lines represent mean and SE (n = 5 except n=4 in two treatments; details are described in the data analysis section).

Stolon treatment did not significantly affect the total flavonoids and chlorogenic acid content in the basal portions (**Table 2**). Earthworms significantly reduced the chlorogenic acid content in the basal portions (**Table 2**; **Figure 4B**). There was a significant interaction between stolon treatment and earthworm treatment on the total flavonoids content in the basal portions. Specifically, when stolons were left intact, earthworms led to a decrease in total flavonoids content, whereas this effect was not observed when stolons were severed (**Table 2**; **Figure 4A**).

**Table 2:** Analysis of variance of the effects of clonal integration, earthworms, and their interaction on total flavonoids and chlorogenic acid content of basal portion, apical portion and whole clonal fragment of *Glechoma longituba*.

Variable	Integration (I)		Earthworm (E)		I × E	
	F_1,14_	*P*	F_1,14_	*P*	F_1,14_	*P*
Basal portion						
Total flavonoids ^a^	3.3	0.093	1.4	0.249	**8.7**	**0.010**
Chlorogenic acid ^a^	0.2	0.646	**14.3**	**0.002**	0.2	0.650
Apical portion						
Total flavonoids ^a^	**5.4**	**0.035**	< 0.1	0.849	< 0.1	0.835
Chlorogenic acid	< 0.1	0.894	1.4	0.257	0.9	0.369
Whole clonal fragment						
Total flavonoids ^a^	**7.5**	**0.016**	0.8	0.383	3.7	0.074
Chlorogenic acid ^a^	< 0.1	0.853	**9.1**	**0.009**	0.8	0.387

Degrees of freedom (d. f.), F and *P* values are given. Values are in bold when *P*<0.05. ^a^Natural log transformation.

In the apical portions, total flavonoids content was significantly higher when stolons were left intact compared to when they were severed (**Table 2**; **Figure 4C**). Neither the presence of earthworms nor its interaction with stolon treatment had a significant effect on the content of total flavonoids or chlorogenic acid in the apical portions (**Table 2**; **Figures 4C, D**).

At the whole fragment level, intact stolons exhibited significantly higher total flavonoids content compared to severed stolons (**Table 2**; **Figure 4E**). Earthworms significantly reduced the chlorogenic acid content at the whole fragment level (**Table 2**; **Figure 4F**). The interaction between stolon treatment and earthworm treatment had no significant effect on content of total flavonoids or chlorogenic acid in the whole fragment (**Table 2**).

### 3.3 Effects of clonal integration and earthworms on soil microbial community of basal and apical portions

A total of 2,266,800 high-quality reads (bacterial 16S rRNA gene) and 2,381,809 reads (fungal ITS1 region) were retained after sequencing and quality filtering from all samples. At the phylum level, Proteobacteria, Acidobacteriota, Actinobacteriota, Chloroflexi, Gemmatimonadota, Planctomycetota, Myxococcota, Bacteroidota, Verrucomicrobiota, and Armatimonadota were identified as the predominant bacterial taxa accounting for 93.8% to 95.6% across different treatments (**Supplementary Figure S1A**). Intact stolons significantly increased the relative abundance of Acidobacteriota in both basal and apical portions but decreased the relative abundance of Actinobacteriota in both basal and apical portions and Gemmatimonadota in basal portions (**Supplementary Tables S1**; **S2**; **Supplementary Figure S1A**). Earthworms significantly increased the relative abundance of Proteobacteria, Bacteroidota, and Verrucomicrobiota in the basal portions, and Acidobacteriota and Verrucomicrobiota in the apical portions. Conversely, they caused a significant decrease in the relative abundance of Actinobacteriota, Gemmatimonadota, and Armatimonadota in the basal portions, and of Actinobacteriota in the apical portions. (**Supplementary Tables S1**; **S2**; **Supplementary Figure S1A**). Furthermore, for basal portions, earthworms significantly increased the relative abundance of Proteobacteria, Planctomycetota, Myxococcota, Bacteroidota, and Verrucomicrobiota while decreasing the relative abundance of Acidobacteriota and Gemmatimonadota when stolons were left intact. However, when stolons were severed, earthworms significantly increased the relative abundance of Acidobacteriota, Bacteroidota, and Verrucomicrobiota but decreased the relative abundance of Proteobacteria, Gemmatimonadota, Planctomycetota and Myxococcota (**Supplementary Tables S1**; **S2**; **Supplementary Figure S1A**). Regarding fungi composition, Ascomycota and Basidiomycota were the most predominant taxa, accounting for 73.9% to 97.1% across different treatments (**Supplementary Figure S1B**). Intact stolons only significantly increased the relative abundance of Glomeromycota in the apical portions (**Supplementary Table S2**; **Supplementary Figure S1B**). The earthworm treatment and its interaction with clonal integration had no significant effect on the fungal community in either the basal or apical portions (**Supplementary Tables S1**; **S2**).

**Figure 5:**
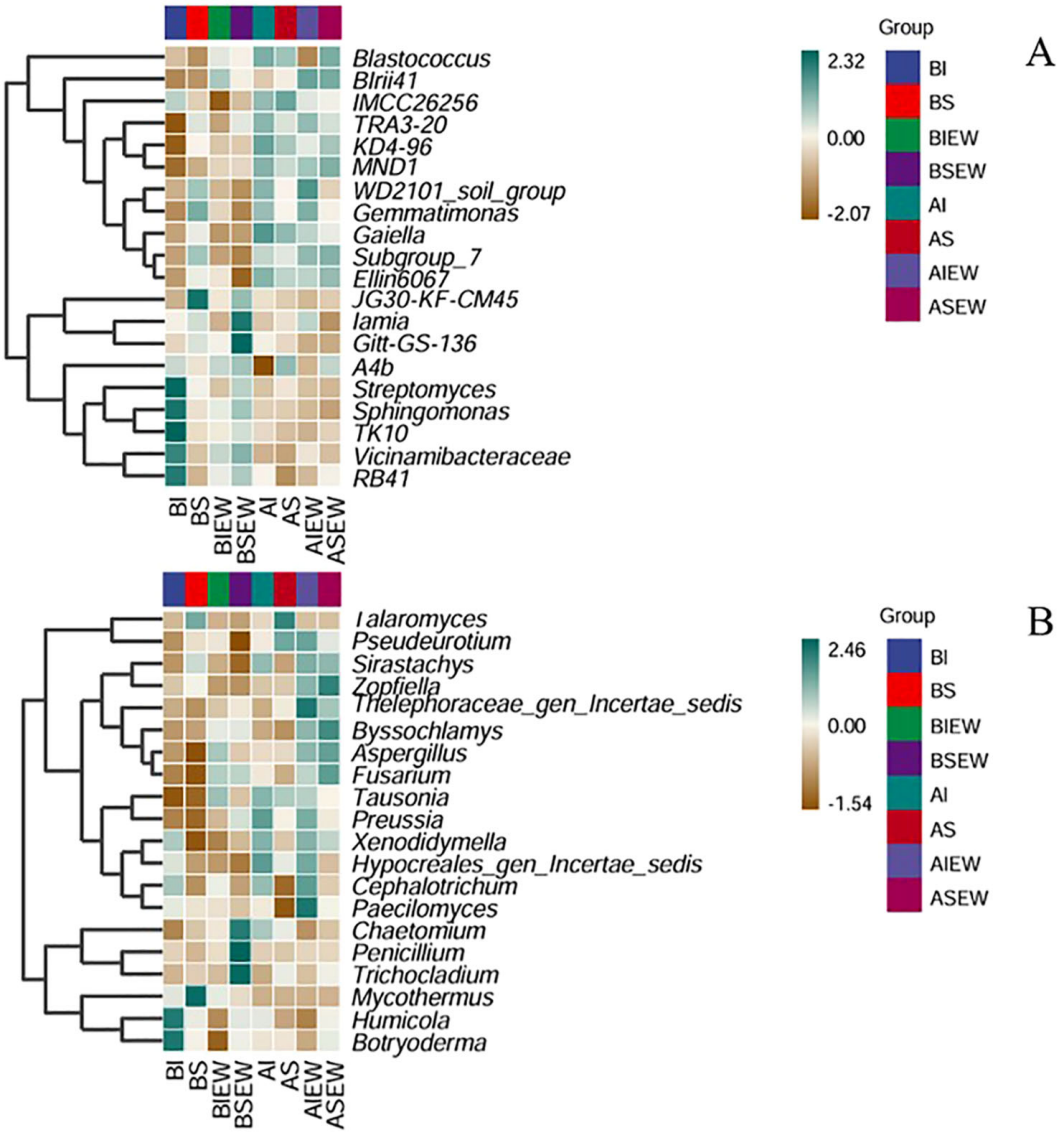
Heatmap for clustering analysis of species abundance of bacterial **(A)** and fungal **(B)** at genus level under different treatments. The green color indicates high values and the brown color indicates low values (n=4). BI: Basal portion-stolons were left intact without Ew; BS: Basal portion-stolons were severed without Ew; BIEw: Basal portion-stolons were left intact with Ew; BSEw: Basal portion-stolons were severed with Ew; AI: Apical portion-stolons were left intact without Ew; AS: Apical portion-stolons were severed without Ew; AIEw: Apical portion-stolons were left intact with Ew; ASEw: Apical portion-stolons were severed with Ew.

The relative abundance of the top 20 genus in the heatmap and the effects of clonal integration and earthworms on the bacterial and fungal communities at the genus level in basal and apical portions are shown in **Figure 5** and **Supplementary Tables S3**-**S6**. The results showed that for basal portions bacteria, clonal integration significantly affected the relative abundance of 9 genera, earthworms significantly affected 5 genera, and their interactions affected 10 genera. For apical portions bacteria, clonal integration significantly affected the relative abundance of 3 genera, earthworms significantly affected 7 genera, and their interactions affected 3 genera. Specifically, intact stolons significantly increased the relative abundance of *Vicinamibacteraceae* and *Sphingomonas* in the basal portion, as well as *Vicinamibacteraceae* and *Iamia* in the apical portions, but decreased the relative abundance of *Iamia* in the basal portions (**Supplementary Tables S3**, **S5**; **Figure 5A**). Earthworms significantly increased the relative abundance of *Vicinamibacteraceae* in the apical portions but decreased the *Gaiella* in the basal portions, and *Sphingomonas* and *Gaiella* of apical portions (**Supplementary Tables S3**, **S5**; **Figure 5A**). For basal portions, when stolons were left intact, earthworms significantly decreased the relative abundance of *Vicinamibacteraceae*, *Sphingomonas*, *Iamia*, and *Streptomyces*, but increased *Iamia* when stolons were severed (**Supplementary Tables S3**, **S5**). Conversely, the *Gemmatimonas* in the basal portions and *Iamia* of apical portions showed opposite trends (**Figure 5A**). For basal portions fungi, clonal integration significantly affected the relative abundance of 2 genera, earthworms significantly affected 7 genera, and their interactions affected 3 genera. For apical portions fungi, clonal integration significantly affected the relative abundance of 2 genera, earthworms significantly affected 2 genera. Specifically, intact stolons significantly decreased the relative abundance of *Trichocladium* and *Chaetomium* of fungal community in the basal portions and *Trichocladium* in the apical portions, but increased the relative abundance of *Cephalotrichum* in the apical portions (**Supplementary Tables S4**, **S6**; **Figure 5B**). Earthworms significantly increased the relative abundance of *Aspergillus*, *Tausonia*, *Fusarium*, and *Chaetomium* in the basal portions and *Fusarium* in the apical portions, while decreasing the relative abundance of *Talaromyces*, *Zopfiella*, and *Botryoderma* in the basal portions and *Talaromyces* in the apical portions (**Supplementary Tables S4**, **S6**; **Figure 5B**). The interaction between clonal integration and earthworm significantly affected the *Humicola*, *Talaromyces*, and *Botryoderma* in the basal portions, (**Supplementary Table S4**). The presence of earthworms resulted in a decrease in the relative abundance of *Talaromyces*, regardless of whether the stolon was intact or severed (**Figure 5B**). Earthworms decreased the relative abundance of *Humicola* and *Botryoderma* when stolons were left intact but increased their relative abundance when stolons were severed (**Figure 5B**).

The differences in soil microbial communities among the different treatments were further investigated using Venn diagrams (**Supplementary Figure S2**). The results showed that intact stolons significantly reduced the number of unique bacterial ASVs in the basal portions (**Supplementary Figure S2A**). In the basal portions, earthworms significantly increased the number of unique bacterial ASVs when stolons were left intact but significantly decreased them when stolons were severed (**Supplementary Figure S2A**). For fungi, earthworms significantly increased the number of unique fungal ASVs in the basal portion, with a more pronounced effect when stolons were intact compared to when they were severed (**Supplementary Figure S2B**).

**Figure 6:**
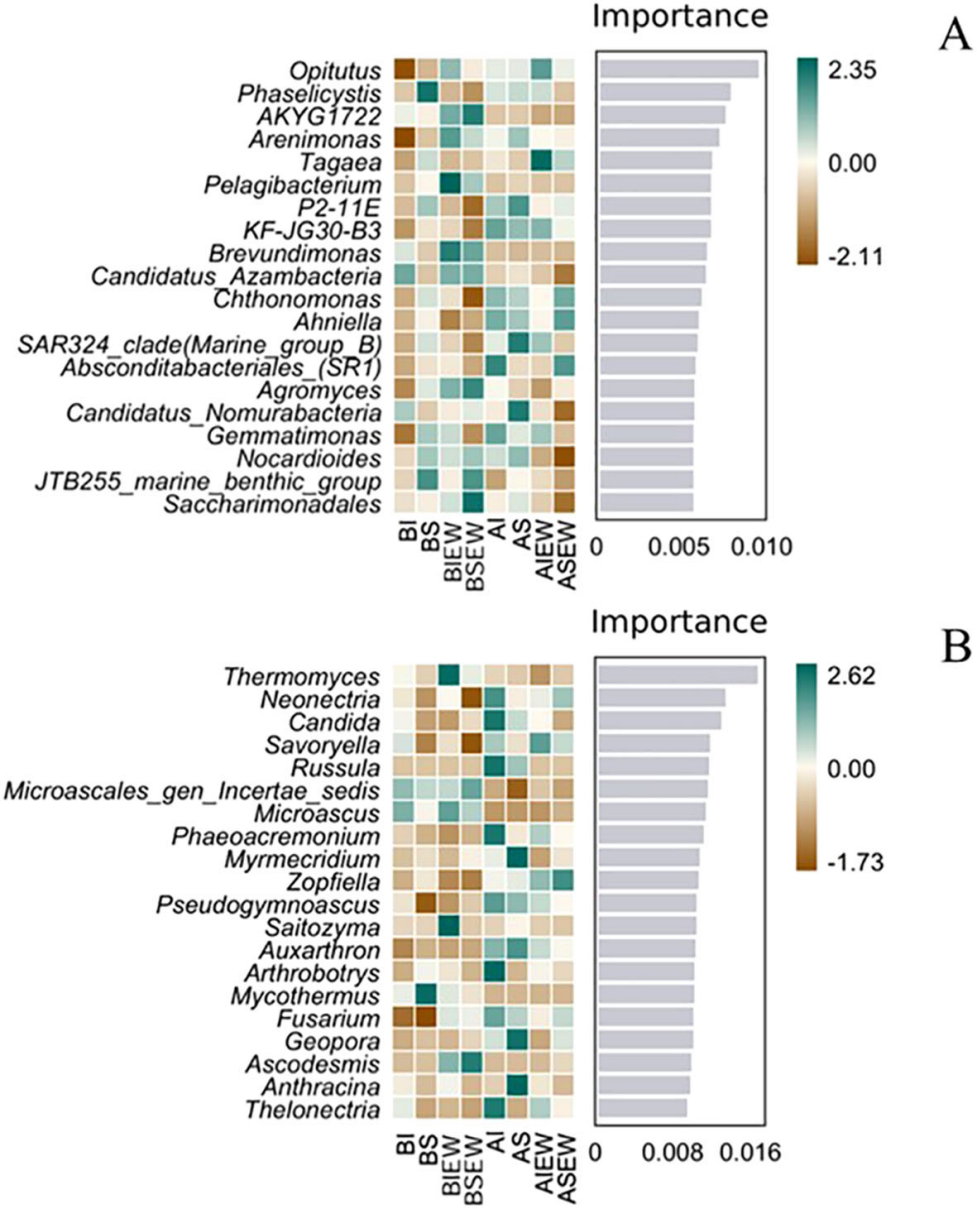
Random-forest analysis of relative abundance and importance various of soil bacteria **(A)** and fungi **(B)** communities at genus level under different treatments (n=4). BI: Basal portion-stolons were left intact without Ew; BS: Basal portion-stolons were severed without Ew; BIEw: Basal portion-stolons were left intact with Ew; BSEw: Basal portion-stolons were severed with Ew; AI: Apical portion-stolons were left intact without Ew; AS: Apical portion-stolons were severed without Ew; AIEw: Apical portion-stolons were left intact with Ew; ASEw: Apical portion-stolons were severed with Ew.

The results of random-forest analysis showed the top 20 importance values of various microbial taxa and their relative abundance across different treatments (**Figure 6**). *Opitutus* had the highest importance value and was the dominant species in the bacterial community across all treatments (**Figure 6A**). Specifically, for both basal and apical portions, *Opitutus* exhibited the highest relative abundance in the treatment involving earthworms when stolons were intact (**Figure 6A**). In the basal portion, the relative abundance of *Arenimonas*, *Pelagibacterium*, *Brevundimonas*, *Candidatus Azambacteria*, *Agromyces*, *Nocardioides*, and *Saccharimonadales* increased in the presence of earthworms. For fungi, *Thermomyces*, *Saitozyma*, and *Ascodesmis* were the dominant species in the basal portion, showing higher relative abundances under the treatment with earthworms when stolons were intact (**Figure 6B**). In the apical portion, *Neonectria*, *Candida*, *Russula*, *Phaeoacremonium*, *Pseudogymnoascus*, *Arthrobotrys*, *Fusarium* and *Thelonectria* were the dominant species, exhibiting higher relative abundances when stolons were intact.

**Figure 7:**
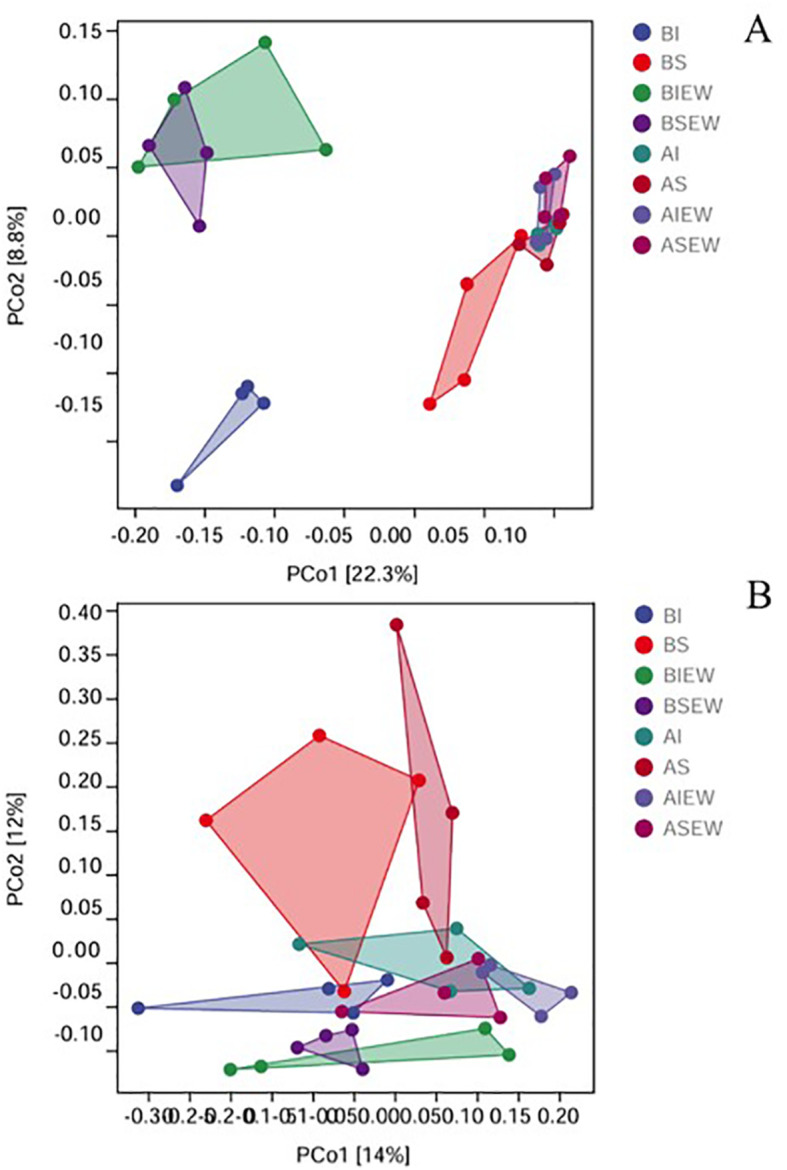
PCoA analysis showing the difference in soil bacterial **(A)** and fungal **(B)** communities between the different treatments (n=4). BI: Basal portion-stolons were left intact without Ew; BS: Basal portion-stolons were severed without Ew; BIEw: Basal portion-stolons were left intact with Ew; BSEw: Basal portion-stolons were severed with Ew; AI: Apical portion-stolons were left intact without Ew; AS: Apical portion-stolons were severed without Ew; AIEw: Apical portion-stolons were left intact with Ew; ASEw: Apical portion-stolons were severed with Ew.

The Principal Coordinate Analysis (PCoA) based on Bray distance further revealed the changes in the structure of bacterial and fungal communities (**Figure 7**). The results demonstrated that treatments with intact stolons and severed stolons were distinctly separated along the first axis for the basal portion, indicating that clonal integration significantly affected the composition of the bacterial community (**Figure 7A**). Additionally, the presence or absence of earthworms in the basal portion was separated along the second axis, while the difference between intact and severed stolon treatments diminished in the presence of earthworms (**Figure 7A**). However, there was no significant difference in the bacterial community structure of the apical portions across different treatments. For fungi, no significant differences were detected among treatments (**Figure 7B**).

**Figure 8:**
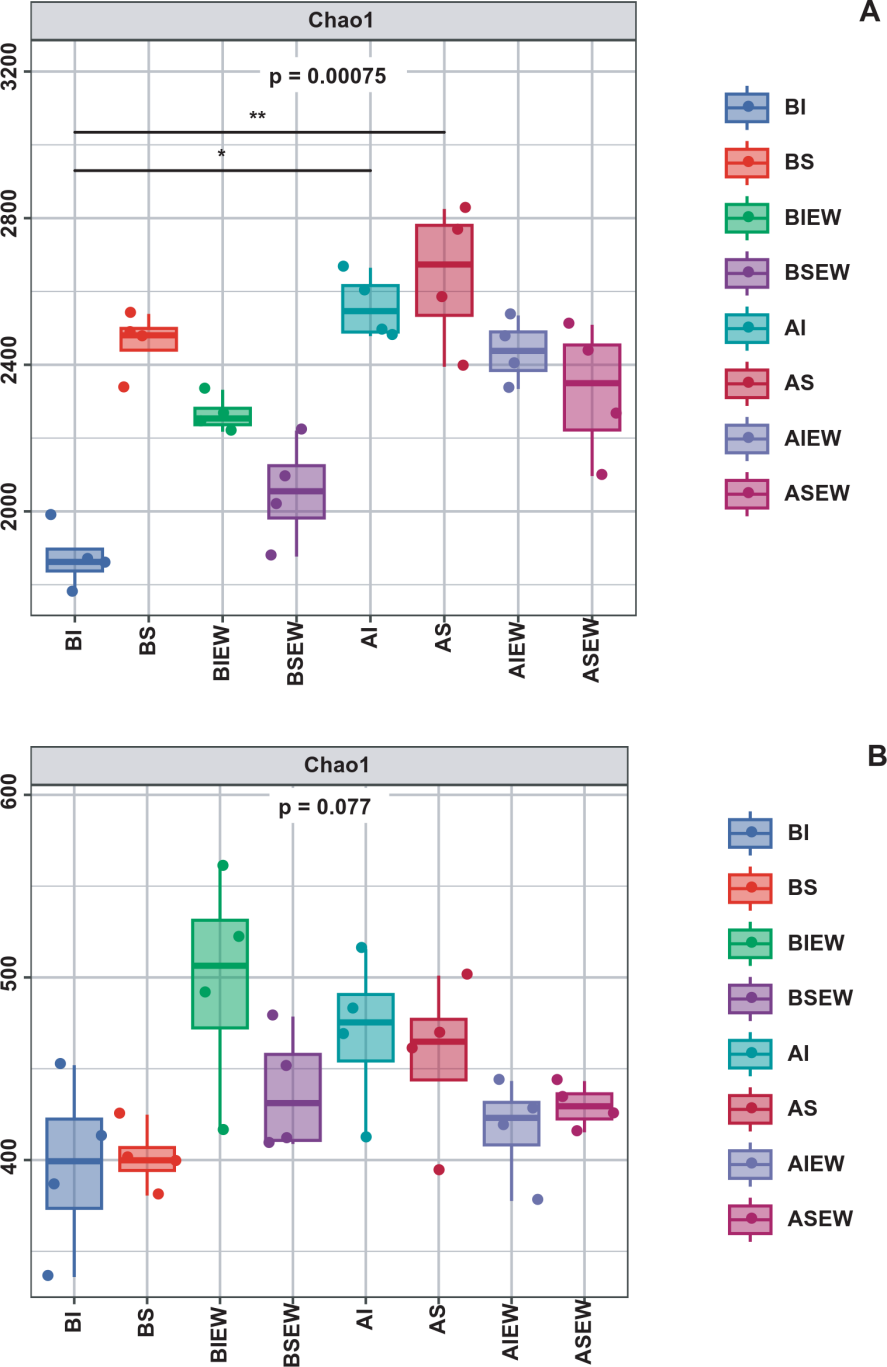
Microbial alpha diversity of bacterial **(A)** and fungal **(B)** communities under different treatment (n=4). BI: Basal portion-stolons were left intact without Ew; BS: Basal portion-stolons were severed without Ew; BIEw: Basal portion-stolons were left intact with Ew; BSEw: Basal portion-stolons were severed with Ew; AI: Apical portion-stolons were left intact without Ew; AS: Apical portion-stolons were severed without Ew; AIEw: Apical portion-stolons were left intact with Ew; ASEw: Apical portion-stolons were severed with Ew.

To assess the effect of earthworms and clonal integration on microbial diversity, the Chao1 richness index was computed (**Supplementary Table S7**; **Figure 8A**). Intact stolons significantly decreased bacterial diversity in the basal portions (**Supplementary Table S7**; **Figure 8A**). Earthworms significantly reduced bacterial diversity in the apical portions. When stolons left intact, the presence of earthworms significantly enhanced bacterial diversity in the basal portions. Conversely, when stolons were severed, bacterial diversity decreased. For fungi, earthworms significantly increased the fungal diversity in the basal portions but significantly decreased it in the apical portions (**Supplementary Table S7**; **Figure 8B**). Clonal integration and its interaction with earthworms had no significant effect on fungal alpha diversity (**Supplementary Table S7**).

**Figure 9:**
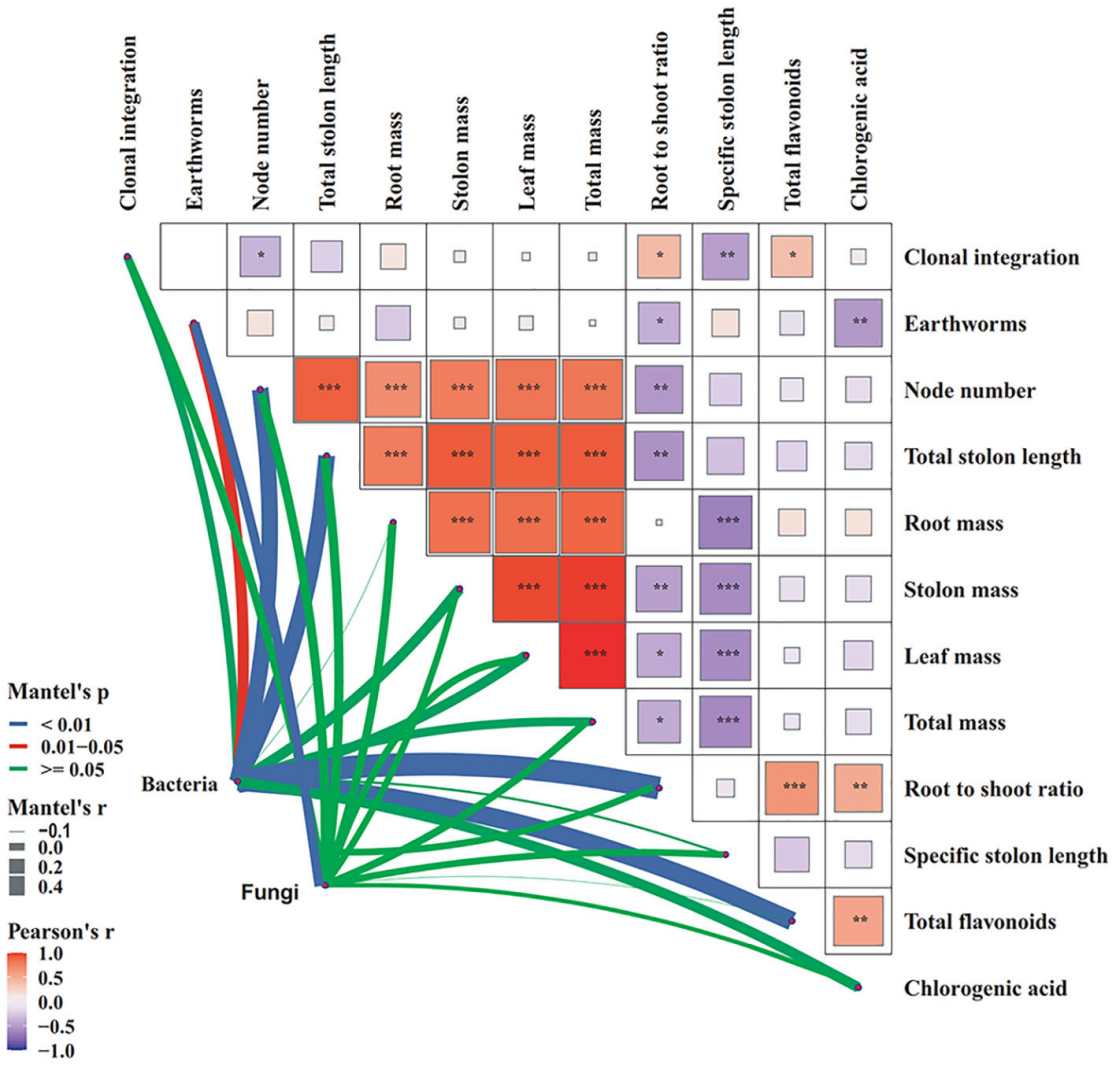
Network heatmap integrating Pearson correlations among plant factors and Mantel test-based associations with bacterial and fungal community structures (n=4).

Multivariate analysis revealed significant associations between microbial communities and plant factors, as well as interrelationships among these plant factors (**Figure 9**). Mantel tests revealed that the assemblages of both bacteria and fungi were significantly influenced by earthworm activity. Moreover, bacterial community composition showed strong correlations with node number, total stolon length, root to shoot ratio, and total flavonoids. Clonal integration was positively correlated with root to shoot ratio and total flavonoids, but negatively correlated with node number and specific stolon length. Notably, earthworm presence exhibited negative associations with root to shoot ratio and chlorogenic acid levels. Additionally, plant chlorogenic acid content was positively linked to total flavonoids.

## 4 Discussion

### 4.1 Response of growth and morphology of *Glechoma longituba* to clonal integration and earthworms

Consistent with some previous studies (Roiloa and Retuerto, 2011; Si et al., 2020; Zhao et al., 2024), leaving the stolon intact reduced basal ramet growth (measured as total biomass) while promoting growth in apical ramets in our experiment. This phenomenon may be attributed to resource translocation from basal ramets to apical ramets through clonal integration, which likely constitutes the primary mechanism underlying the differential growth allocation between the basal and apical portions in *G. longituba*. At the whole fragment level, total growth remained unchanged, indicating that clonal integration enhances resource optimization rather than increasing net biomass production in *G. longituba*. The responses of node number and total stem length at both the basal and apical portions similar as those of total biomass; however, these measurements were notably reduced at the whole fragment level. These findings suggest that clonal integration may inhibit the production and dispersal of clonal offspring in *G. longituba*, as vegetative propagation occurs through node development and longer stems offer greater potential for generating more nodes and distributing clonal offsprings over a wider growth area (Chu et al., 2006; Zhang et al., 2023).

The effect of earthworms on the basal portions was primarily reflected in biomass allocation. Specifically, the presence of earthworms reduced root investment in the basal portions of *G. longituba*. At the whole fragment level, earthworms significantly increased the number of nodes. These effects were primarily attributed to earthworm burrowing activity, which homogenized soil structure, enhanced nutrient availability, and directed biomass allocation toward aboveground organs (Roiloa and Retuerto, 2007). This shift likely optimizes resource acquisition and stimulates node formation.

Crucially, earthworms enhanced the benefits of clonal integration in apical ramets, significantly promoting node formation when stolons were left intact compared to when earthworms were absent. At the whole fragment level, earthworms exerted a substantial positive effect, alleviating the negative impacts of clonal integration on both node number and total stem length. These findings demonstrate interactive influence of earthworms and clonal integration on the potential for asexual reproduction.

### 4.2 Response of content of the total flavonoids and chlorogenic acid of *Glechoma longituba* to clonal integration and earthworms

Flavonoids and chlorogenic acid represent primary bioactive constituents in *G. longituba* (Jin et al., 2019b; Zhang et al., 2012), valued for their antioxidant and anti-inflammatory properties (Zhang et al., 2012; Jin et al., 2019b). Clonal integration significantly enhanced total flavonoid accumulation both in apical ramets and at the whole fragment level, largely due to improved resource allocation toward flavonoid synthesis in resource-receiving apical ramets. In contrast, earthworms suppressed flavonoid accumulation in basal ramets. This suppression likely resulted from earthworm-driven alterations in soil nitrogen dynamics that earthworms enhance soil nitrogen availability, thereby triggering a reallocation of carbon and nitrogen resources in plants to prioritize growth requirements (van Groenigen et al., 2014; Kim et al., 2022; Ratsiatosika et al., 2024). This shift leads to the transcriptional downregulation of key enzymes in the phenylpropanoid pathway, ultimately resulting in reduced flavonoid synthesis (Blount et al., 2000; He et al., 2024; Zhang et al., 2025). The reduction of chlorogenic acid in basal ramets and whole fragments was primarily attributed to earthworm activity, potentially via microbial degradation of phenolic compounds or altered expression of hydroxycinnamoyl-CoA quinate transferase genes (Moglia et al., 2014; Payyavula et al., 2015; Cardenas et al., 2021).

Notably, neither flavonoids nor chlorogenic acid in apical ramets showed significant responses to earthworm presence, indicating that soil chemistry predominantly governs metabolite production in basal ramets, whereas clonal integration regulates accumulation in apical ramets. These findings demonstrate that earthworm activity may compromise the medicinal quality *G. longituba* by disrupting secondary metabolite accumulation, highlighting the need for strategic earthworm management in cultivation systems.

### 4.3 Response of microbial community of *Glechoma longituba* to clonal integration and earthworms

Previous studies have demonstrated that clonal integration affected soil microbial activity by influencing carbon availability, nitrogen mineralization and nitrification, and altering microbial biomass (Butler et al., 2004; Chen et al., 2015; Ma et al., 2023). Earthworms are known to independently affect soil microbial communities through priming effects and signal molecules (Aghababaei et al., 2014; Binet et al., 1998; Lavelle et al., 2016). In this study, we investigated the effect of clonal integration and earthworms on soil microbial communities in the root zone of *G. longituba*, revealing distinct response patterns between bacteria and fungi.

Our results demonstrated that clonal integration significantly affected the composition of bacterial and fungal communities in both basal and apical portions. As a key evolutionary adaptation of clonal plants, clonal integration enables the transfer and sharing of essential resources (such as photosynthates, water, and mineral nutrients) between connected ramets (Hutchings and de Kroon, 1994; Kroon, 1993; Song et al., 2013). This process not only affects the plants growth but also significantly alters the belowground micro-environment, thereby influencing the structure and function of microbial communities of specific microbial species (Li et al., 2018; Zhao et al., 2025). Changes in soil microbial communities are intrinsically associated with the physiological status of plants, reflecting a dynamic and reciprocal interaction between the two (Berendsen et al., 2012). Our data further revealed strong correlations between plant performance indicators and the composition of microbial communities. Specifically, clonal integration significantly decreased the relative abundance of *Iamia* in bacterial community in the basal portions but increased the relative abundance of *Iamia* in the apical portions. Research has shown that *Iamia*, belonging to the phylum Actinobacteria, possesses the capacity to synthesize plant growth hormones and enhance plant stress tolerance (Kavya et al., 2024; Kurahashi et al., 2009; Selim et al., 2019). Moreover, it exhibits a close correlation with the growth status of plants. The variations in the abundance of *Iamia* might be associated with the resource allocation adjustments between the basal and apical portions via clonal integration. Our findings indicate that clonal integration promotes the growth of apical portions while consuming resources from the basal portions. Similar trends were observed for fungi, where clonal integration notably augmented the relative abundance of dominant species in apical portions. Furthermore, clonal integration had a greater effect on bacteria than on fungi. Bacteria possess relatively simple cell structures and exhibit high reproductive rates, enabling them to respond more rapidly to resource alterations and signal transduction induced by clonal integration (Miller and Bassler, 2001; West et al., 2006). This leads to more pronounced changes in the structure and function of the bacterial community. Conversely, fungi have more complex cell structures, longer growth cycles, and slower responses to environmental changes, resulting in a relatively subdued impact of clonal integration on these organisms (Boer et al., 2005; Kohn, 2005).

Our research indicated that earthworms significantly affected the composition and diversity of bacterial and fungal communities in both basal and apical portions. The introduction of earthworms reduced bacterial diversity and the number of unique bacterial species, whereas it increased fungal diversity and the presence of distinct fungal species. In contrast to bacterial communities, fungal community composition was significantly correlated with the presence of earthworms alone, and not with any measured plant growth indicators. These findings are consistent with a previous study that have shown earthworms enrich specific microbial communities (Wang et al., 2024b). For example, *Opitutus*, identified as a dominant species, was significantly enriched after the addition of earthworms, which significantly influenced the structure of the microbial community. *Opitutus* may participate in complex material cycling and metabolic processes in the soil, affecting nutrient availability (Yi et al., 2019). When compared to the effect of clonal integration, fungi were more significantly influenced by earthworms. As important soil ecosystem engineers, earthworms can directly alter the physical structure and chemical properties of soil through activities such as feeding, excretion, and burrowing (Lavelle and Spain, 2024; Wu et al., 2025). Consequently, they create either more suitable or challenging living environments for fungi, making the impact of earthworms on fungi particularly remarkable (Nechitaylo et al., 2010; Song et al., 2020; Wang et al., 2024b),. Additionally, earthworms had a further effect on the bacteria and fungi at the basal and apical portions through clonal integration. Earthworms reduced the differences in bacterial communities between intact and severed stolon treatments in the basal portions. Moreover, earthworms exhibited opposite trends of influence in the intact and severed treatments. By altering the physical structure and chemical properties of the soil through activities like feeding, excreting, and burrowing, earthworms may homogenize the soil environment (Wu et al., 2025). This caused bacterial communities that originally differed due to different stolon connection states to gradually become more similar.

## 5 Conclusion

Clonal integration did not enhance overall growth of *G. longituba*, suggesting that it may not increase production yields. However, it significantly promotes the accumulation of total flavonoids at the whole fragment level. Although earthworms did not significantly affect overall yield, they mitigated the inhibitory effects of clonal integration on node formation and stolon elongation. Notably, the presence of earthworms substantially decreased the accumulation of chlorogenic acid at the whole fragment level. Furthermore, our research indicated that both clonal integration and earthworms significantly affected the composition and diversity of bacterial and fungal communities in basal and apical. Bacteria and fungi responded differently to these factors: bacteria were more strongly influenced by clonal integration, while fungi were more significantly impacted by earthworms. To some extent, earthworms mitigated the disparities induced by clonal integration, leading to a more homogenized microbial community structure. These findings offer valuable insights into the cultivation strategies for clonal plants, particularly those with significant economic and medicinal importance.

## Data Availability

The datasets presented in this study can be found in online repositories. The names of the repository/repositories and accession number(s) can be found below: https://www.ncbi.nlm.nih.gov/, PRJNA1235935.
