# Supplementary material for: The effects of clonal integration and earthworms on the growth, active constituent accumulation in Glechoma longituba, and the soil microbial community in its root zone
Source: Front Plant Sci. 2025 Sep 15;16:1596905. doi: 10.3389/fpls.2025.1596905 (PMC12477172; doi:10.3389/fpls.2025.1596905)
Supplement: Supplementary file 1 [file DataSheet1.pdf]

Supplementary Material

Table S1. Analysis of variance of the effects of clonal integration, earthworms, and their interaction on composition of bacterial and fungal communities at phylum level in root zone soil of basal portion of *Glechoma longituba*. Degree of freedom (subscript for "F"), F and *P* values are given. Values are in bold when $P < 0.05$.

Variable	Integration (I)		Earthworm (E)		I × E	
	$F_{1, 12}$	*P*	$F_{1, 12}$	*P*	$F_{1, 12}$	*P*
Bacterial community						
Proteobacteria [a]	< 0.1	0.945	**4.8**	**0.049**	**5.6**	**0.035**
Acidobacteriota [a]	**22.4**	**< 0.001**	< 0.1	0.944	**27.6**	**< 0.001**
Actinobacteriota [a]	**5.2**	**0.042**	**11.3**	**0.006**	0.2	0.686
Chloroflexi	0.6	0.448	1.6	0.235	0.5	0.492
Gemmatimonadota	**9.8**	**0.009**	**10.8**	**0.007**	**5.2**	**0.041**
Planctomycetota [a]	4.3	0.060	0.5	0.492	**5.5**	**0.037**
Myxococcota [a]	0.2	0.663	0.6	0.470	**7.2**	**0.020**
Bacteroidota [a]	< 0.1	0.935	**28.6**	**< 0.001**	**5.2**	**0.041**
Verrucomicrobiota [a]	3.6	0.082	**30.1**	**< 0.001**	**13.3**	**0.003**
Armatimonadota [b]	2.3	0.159	**6.9**	**0.022**	2.6	0.135
**Fungal community**						
Ascomycota	0.3	0.599	< 0.1	0.961	2.0	0.187
Basidiomycota	1.2	0.301	2.9	0.113	3.3	0.094
Mortierellomycota [a]	0.3	0.588	2.2	0.160	1.9	0.196
Chytridiomycota [b]	< 0.1	0.888	0.3	0.576	4.0	0.069
Rozellomycota	1.5	0.251	1.6	0.224	1.5	0.244
Aphelidiomycota [b]	0.9	0.372	0.0	0.799	< 0.1	0.842
Mucoromycota	2.9	0.114	2.9	0.114	2.9	0.114
Glomeromycota [b]	0.6	0.448	0.1	0.750	0.4	0.561
Kickxellomycota	1.0	0.337	1.0	0.337	1.0	0.337

[a] Natural log transformation. [b] Square root transformation.

Table S2. Analysis of variance of the effects of clonal integration, earthworms, and their interaction on composition of bacterial and fungal communities at phylum level in root zone soil of apical portion of *Glechoma longituba*. Degree of freedom (subscript for "F"), F and *P* values are given. Values are in bold when $P < 0.05$.

Variable	Integration (I)		Earthworm (E)		I × E	
	$F_{1, 12}$	*P*	$F_{1, 12}$	*P*	$F_{1, 12}$	*P*
Bacterial community						
Proteobacteria [a]	< 0.1	0.758	< 0.1	0.847	0.4	0.529
Acidobacteriota	0.7	0.420	**7.4**	**0.019**	2.6	0.132
Actinobacteriota	0.3	0.577	**7.2**	**0.020**	0.7	0.415
Chloroflexi [a]	3.3	0.093	2.2	0.164	3.0	0.106
Gemmatimonadota [a]	0.7	0.408	0.6	0.444	0.2	0.648
Planctomycetota [a]	< 0.1	0.845	4.8	0.050	1.0	0.341
Myxococcota [a]	< 0.1	0.890	3.8	0.074	4.6	0.054
Bacteroidota [a]	0.5	0.477	< 0.1	0.927	2.4	0.145
Verrucomicrobiota [a]	0.2	0.654	**5.0**	**0.045**	< 0.1	0.784
Armatimonadota [a]	0.2	0.661	0.6	0.468	3.6	0.082
Fungal community						
Ascomycota	0.4	0.520	< 0.1	0.797	< 0.1	0.973
Basidiomycota	0.2	0.678	0.5	0.513	0.2	0.630
Mortierellomycota [a]	< 0.1	0.986	< 0.1	0.836	< 0.1	0.936
Chytridiomycota	0.4	0.516	0.5	0.489	0.8	0.388
Rozellomycota	2.9	0.112	2.9	0.112	2.9	0.112
Aphelidiomycota [b]	< 0.1	0.937	0.7	0.420	0.7	0.412
Glomeromycota	**4.9**	**0.048**	1.4	0.257	0.7	0.422

[a] Natural log transformation. [b] Square root transformation.

Table S3. Analysis of variance of the effects of clonal integration, earthworms, and their interaction on composition of bacterial community at genus level in root zone soil of basal portion of *Glechoma longituba*. Degree of freedom (subscript for "F"), F and *P* values are given. Values are in bold when $P < 0.05$.

Variable	Integration (I)		Earthworm (E)		I × E	
	$F_{1, 12}$	*P*	$F_{1, 12}$	*P*	$F_{1, 12}$	*P*
Bacterial community						
Vicinamibacteraceae [a]	**5.1**	**0.044**	0.4	0.541	**14.5**	**0.002**
Sphingomonas	**10.1**	**0.008**	2.8	0.122	**30.1**	**< 0.001**
KD4-96 [a]	**13.8**	**0.003**	2.7	0.127	**12.2**	**0.004**
RB41 [b]	**6.2**	**0.028**	0.1	0.753	**15.0**	**0.002**
MND1 [a]	1.8	0.205	**5.6**	**0.036**	1.4	0.257
JG30-KF-CM45 [a]	**8.5**	**0.013**	< 0.1	0.858	1.5	0.240
Blastococcus [a]	0.2	0.698	0.3	0.617	< 0.1	0.984
Blrii41 [a]	0.1	0.765	**7.3**	**0.019**	0.5	0.514
A4b [b]	< 0.1	0.987	0.3	0.608	< 0.1	0.848
TK10 [a]	2.9	0.113	2.1	0.175	**6.9**	**0.022**
TRA3-20 [b]	**17.6**	**0.001**	1.2	0.286	1.6	0.237
IMCC26256 [a]	< 0.1	0.854	2.2	0.164	1.7	0.213
Iamia [a]	**16.3**	**0.002**	0.3	0.608	**8.6**	**0.013**
WD2101_soil_group	0.3	0.584	0.9	0.354	1.1	0.314
Streptomyces [a]	0.3	0.576	4.1	0.066	**9.1**	**0.011**
Gitt-GS-136 [a]	**12.4**	**0.004**	**7.7**	**0.017**	2.6	0.136
Gaiella [a]	4.3	0.061	**5.9**	**0.032**	2.7	0.124
Subgroup_7	**13.1**	**0.004**	**33.2**	**< 0.001**	**33.2**	**< 0.001**
Ellin6067 [a]	0.2	0.669	3.2	0.098	**11.0**	**0.006**
Gemmatimonas [a]	2.1	0.169	3.2	0.098	**13.2**	**0.003**

[a] Natural log transformation. [b] Square root transformation.

Table S4. Analysis of variance of the effects of clonal integration, earthworms, and their interaction on fungal community composition at genus level in root zone soil of basal portion of *Glechoma longituba*. Degree of freedom (subscript for "F"), F and *P* values are given. Values are in bold when *P*＜0.05.

Variable	Integration (I)		Earthworm (E)		I × E	
	$F_{1, 12}$	*P*	$F_{1, 12}$	*P*	$F_{1, 12}$	*P*
Fungal community						
Humicola [b]	< 0.1	0.814	3.7	0.080	**5.6**	**0.036**
Aspergillus [a]	2.8	0.119	**7.3**	**0.019**	< 0.1	0.791
Talaromyces [a]	3.4	0.089	**18.6**	**0.001**	**17.7**	**0.001**
Tausonia [a]	0.2	0.637	**5.4**	**0.039**	0.9	0.363
Xenodidymella	0.8	0.384	0.2	0.649	2.1	0.172
Fusarium [a]	0.2	0.638	**11.9**	**0.005**	< 0.1	0.786
Preussia [a]	< 0.1	0.947	2.6	0.131	< 0.1	0.789
Byssochlamys	< 0.1	0.931	1.5	0.250	< 0.1	0.982
Penicillium	1.7	0.217	2.8	0.122	2.4	0.147
Sirastachys [a]	< 0.1	0.921	< 0.1	0.863	0.2	0.645
Zopfiella [b]	0.6	0.448	**12.4**	**0.004**	1.6	0.228
Cephalotrichum [a]	4.3	0.059	0.4	0.550	0.5	0.493
Thelephoraceae_gen_ Incertae_sedis	< 0.1	0.953	0.8	0.396	0.2	0.644
Paecilomyces	0.6	0.460	0.6	0.438	< 0.1	0.907
Trichocladium [a]	**7.5**	**0.018**	1.7	0.217	1.6	0.236
Mycothermus [a]	0.5	0.493	3.6	0.083	1.1	0.320
Hypocreales_gen_ Incertae_sedis	1.6	0.226	1.8	0.207	0.5	0.477
Botryoderma [b]	0.3	0.598	**12.5**	**0.004**	**13.7**	**0.003**
Pseudeurotium [a]	< 0.1	0.833	0.1	0.712	1.9	0.198
Chaetomium [b]	**7.5**	**0.018**	**5.7**	**0.035**	0.7	0.413

[a] Natural log transformation. [b] Square root transformation.

Table S5. Analysis of variance of the effects of clonal integration, earthworms, and their interaction on composition of bacterial community at genus level in root zone soil of apical portion of *Glechoma longituba*. Degree of freedom (subscript for "F"), F and *P* values are given. Values are in bold when $P < 0.05$.

Variable	Integration (I)		Earthworm (E)		I × E	
	$F_{1, 12}$	*P*	$F_{1, 12}$	*P*	$F_{1, 12}$	*P*
Bacterial community						
Vicinamibacteraceae	**6.6**	**0.025**	**14.7**	**0.002**	1.2	0.301
Sphingomonas	1.9	0.195	**10.7**	**0.007**	0.9	0.349
KD-496	< 0.1	0.955	**7.9**	**0.016**	**8.3**	**0.014**
RB41 [a]	0.4	0.552	0.4	0.525	4.6	0.054
MND1	0.3	0.613	0.4	0.518	2.6	0.132
JG30-KF-CM45 [a]	<0.1	0.867	0.3	0.618	0.3	0.621
Blastococcus	1.5	0.252	1.4	0.262	2.4	0.145
Blrii41 [a]	0.3	0.584	**11.3**	**0.006**	0.5	0.509
A4b [a]	2.0	0.181	0.5	0.486	0.1	0.735
TK10	<0.1	0.927	< 0.1	0.772	0.7	0.411
TRA3-20 [a]	1.0	0.340	<0.1	0.983	<0.1	0.876
IMCC26256	< 0.1	0.823	4.1	0.065	0.3	0.589
Iamia [a]	**4.9**	**0.047**	<0.1	0.861	**10.7**	**0.007**
WD2101_soil_group	**10.9**	**0.006**	<0.1	0.914	1.0	0.326
Streptomyces	0.2	0.681	0.7	0.417	0.5	0.482
Gitt-GS-136 [a]	0.6	0.467	**10.9**	**0.006**	0.3	0.569
Gaiella	2.6	0.133	**9.0**	**0.011**	0.1	0.768
Subgroup_7	< 0.1	0.792	**5.8**	**0.033**	0.6	0.450
Ellin6067 [a]	0.1	0.712	0.1	0.756	**6.9**	**0.022**
Gemmatimonas	4.4	0.057	<0.1	0.833	< 0.1	0.761

[a] Natural log transformation.

Table S6. Analysis of variance of the effects of clonal integration, earthworms, and their interaction on composition of fungal community at genus level in root zone soil of apical portion of *Glechoma longituba*. Degree of freedom (subscript for "F"), F and *P* values are given. Values are in bold when $P < 0.05$.

Variable	Integration (I)		Earthworm (E)		I × E	
	$F_{1, 12}$	*P*	$F_{1, 12}$	*P*	$F_{1, 12}$	*P*
Fungal community						
Humicola [b]	0.1	0.710	0.1	0.721	3.7	0.078
Aspergillus	< 0.1	0.819	3.3	0.095	< 0.1	0.795
Talaromyces [a]	2.3	0.153	**5.3**	**0.040**	2.4	0.151
Tausonia	0.2	0.669	0.2	0.627	< 0.1	0.922
Xenodidymella [b]	1.7	0.212	0.6	0.458	0.4	0.535
Fusarium [a]	0.1	0.812	**5.2**	**0.042**	1.1	0.326
Preussia [a]	1.1	0.311	0.4	0.520	0.2	0.664
Byssochlamys [a]	< 0.1	0.958	4.7	0.051	0.1	0.757
Penicillium	0.1	0.761	1.2	0.303	0.2	0.677
Sirastachys	1.6	0.232	1.8	0.202	1.1	0.313
Zopfiella [a]	0.2	0.651	2.5	0.137	< 0.1	0.765
Cephalotrichum	**7.7**	**0.017**	1.0	0.342	< 0.1	0.816
Thelephoraceae_gen _Incertae_sedis [a]	< 0.1	0.886	3.9	0.073	0.8	0.378
Paecilomyces [a]	2.6	0.130	4.0	0.068	< 0.1	0.861
Trichocladium [a]	**6.0**	**0.030**	0.8	0.389	< 0.1	0.871
Mycothermus	1.9	0.190	< 0.1	0.962	1.1	0.312
Hypocreales_gen_ Incertae_sedis	4.5	0.055	0.3	0.565	< 0.1	0.771
Botryoderma	1.3	0.275	< 0.1	0.766	1.3	0.278
Pseudeurotium [a]	< 0.1	0.955	0.1	0.722	1.0	0.345
Chaetomium [a]	0.3	0.568	4.1	0.065	1.2	0.294

[a] Natural log transformation. [b] Square root transformation.

Table S7. Analysis of variance of the effects of clonal integration, earthworms, and their interaction on bacterial and fungal alpha diversity in root zone soil of basal portion and apical portion of *Glechoma longituba*. Degree of freedom (subscript for "F"), F and *P* values are given. Values are in bold when $P < 0.05$.

Variable	Integration (I)		Earthworm (E)		I × E	
	$F_{1, 12}$	*P*	$F_{1, 12}$	*P*	$F_{1, 12}$	*P*
**Basal portion**						
Bacterial diversity						
Chao1	**14.7**	**0.002**	< 0.1	0.878	**67.1**	**< 0.001**
Fungal diversity						
Chao1 [a]	1.2	0.300	**9.8**	**0.009**	2.0	0.182
**Apical portion**						
Bacterial diversity						
Chao1 [a]	< 0.1	0.772	**8.6**	**0.013**	1.7	0.219
Fungal diversity						
Chao1 [a]	< 0.1	0.984	**4.9**	**0.047**	0.6	0.456

[a] Natural log transformation.

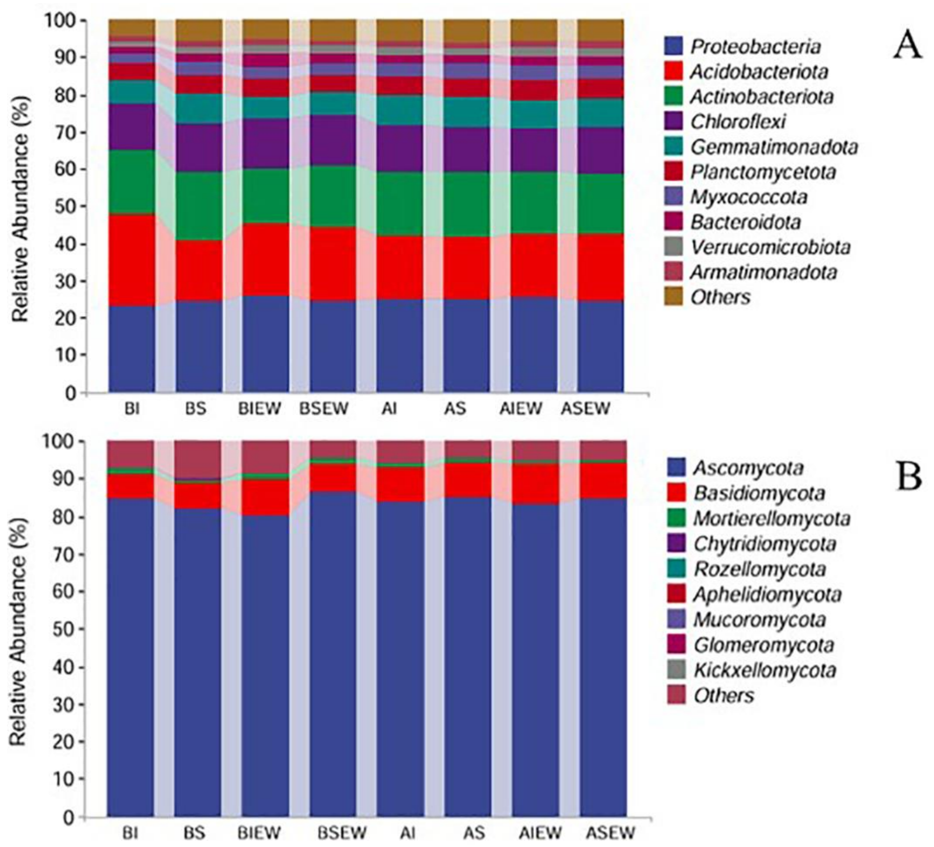

Figure S1 The composition of bacterial (A) and fungal (B) communities at phyla level under different treatments (n=4). BI: Basal portion-stolons were left intact without Ew; BS: Basal portion-stolons were severed without Ew; BIEw: Basal portion-stolons were left intact with Ew; BSEw: Basal portion-stolons were severed with Ew; AI: Apical portion-stolons were left intact without Ew; AS: Apical portion-stolons were severed without Ew; AIEw: Apical portion-stolons were left intact with Ew; ASEw: Apical portion-stolons were severed with Ew.

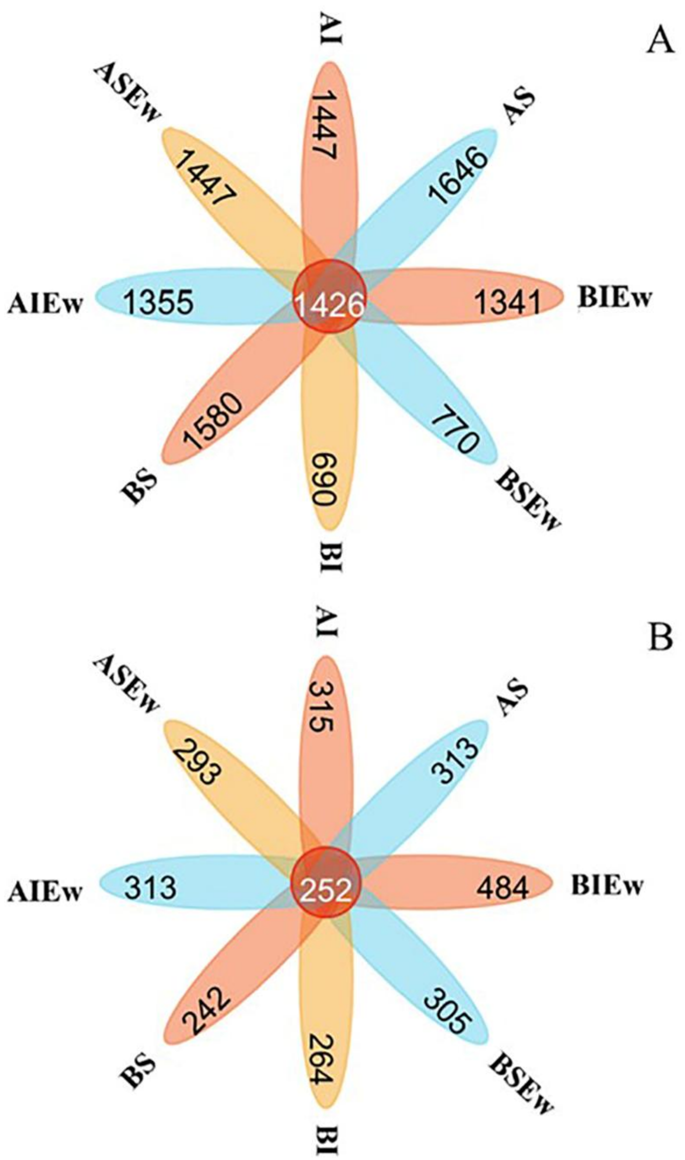

Figure S2 Venn diagrams showing the number of shared and unique OTUs of bacterial (A) and fungal (B) communities between different treatments (n=4). BI: Basal portion-stolons were left intact without Ew; BS: Basal portion-stolons were severed without Ew; BIEw: Basal portion-stolons were left intact with Ew; BSEw: Basal portion-stolons were severed with Ew; AI: Apical portion-stolons were left intact without Ew; AS: Apical portion-stolons were severed without Ew; AIEw: Apical portion-stolons were left intact with Ew; ASEw: Apical portion-stolons were severed with Ew.
